# AKI Epidemiology and Outcomes: A Retrospective Cohort Study from the Prenephrology Era

**DOI:** 10.1155/2021/5549316

**Published:** 2021-04-26

**Authors:** K. Asmus, S. Erfurt, O. Ritter, S. Patschan, D. Patschan

**Affiliations:** Zentrum für Innere Medizin 1, Kardiologie, Angiologie, Nephrologie, Klinikum Brandenburg, Medizinische Hochschule Brandenburg, Brandenburg, Germany

## Abstract

**Background:**

Acute kidney injury substantially worsens the prognosis of hospitalized patients. The Brandenburg Medical School was founded in 2014, and a nephrology section was opened in summer 2017. The aim of the study was to analyze AKI epidemiology and outcomes in one of two university hospitals belonging to the medical school. The period of interest dated from January to December 2015.

**Methods:**

The investigation was designed as a single-center, retrospective cohort study at the Brandenburg Hospital of the Brandenburg Medical School. All in-hospital patients treated between January and the end of December 2015 were included. AKI was defined as specified in the 2012 published KDIGO criteria (criteria 1 and 2). Four parameters were evaluated in particular: AKI incidence, in-hospital mortality, frequency of renal replacement therapy, and renal recovery during the stay at the hospital.

**Results:**

A total number of 5,300 patients were included in the analysis. AKI was diagnosed in 490 subjects (10.1%). The in-hospital mortality was 26%. The following conditions/parameters significantly differed between survivors (s) and nonsurviving (ns) subjects: duration of in-hospital treatment (s > ns), AKI onset (outpatient vs. in-hospital) (outpatient in s > ns), dialysis due to AKI (s < ns), vasopressor administration (*s* < ns), and invasive ventilation (s < ns). 5.6% received dialysis therapy, and renal recovery occurred in 31% of all surviving AKI subjects.

**Conclusion:**

Both, the AKI incidence and the frequency of dialysis were lower than reported in the literature. However, fewer subjects recovered from AKI. These discrepant findings possibly result from the lack of prehospitalization creatinine values, the lack of follow-up data, and a generally lower awareness for the need to perform renal replacement therapy in AKI.

## 1. Introduction

Acute kidney injury (AKI) affects increasing numbers of in-hospital patients in Central Europe and the US. In recent years, the incidence of the syndrome has significantly been increased [1]. Relevant causes are aging in general and higher average morbidity which both increase the susceptibility to diagnostic and therapeutic procedures [2]. Nevertheless, the prognosis of AKI remains poor since almost 25 years. Some progress has been achieved in terms of early AKI diagnosis [3], and limited progress has, however, been achieved with regard to AKI management [4, 5]. At the intensive care unit (ICU), mortality of affected subjects reaches 50% or even more, depending on the circumstances which aggravate kidney dysfunction/induce AKI [4]. AKI has been identified as an independent predictor of mortality at the ICU [6]. Therapeutic measures aim to prevent the kidney from further damage [7], and specific interventions, particularly those that may promote renal recovery, are missing in most situations. Therefore, nephrologists all over the world strive to identify more effective strategies for early AKI diagnosis and a more sophisticated therapy.

Despite AKI has been recognized as frequent and serious complication in German hospitals since a long time, epidemiological data on AKI incidence and outcomes in Germany are still limited. In 2019, Khadzhynov and colleagues [8] published a retrospective study which included more than 180,000 individuals (period of 3.5 years). The authors identified a cumulative incidence of 21.4% (sum of AKIN stages 1–3) with an increasing mortality risk from AKIN stages 1–3. The data were acquired in the Charité Medical Center, one of the largest university hospitals in the whole country. Topographically, the city of Berlin is embedded in the state of Brandenburg. Both, Berlin and Brandenburg are, however, individual federal states of Germany. In 2014, the Brandenburg Medical School was founded as first university of medicine in the whole state. Its fundamental mission is to improve the quality of healthcare in rural areas around the capital. In 2017, finally, a new department of nephrology was opened as part of the Center of Internal Medicine 1 (cardiology–angiology–nephrology). It was the first time since the mid-1990s that a nephrologist in responsibility was hired in order to improve the care of patients with kidney diseases. In the current investigation, which was designed as a retrospective cohort study, we evaluated epidemiological data of patients treated at the Brandenburg Hospital between January and the end of December 2015. We aimed to analyze the situation prior to 2017 in a representative manner. The ultimate goal was to characterize the nephrology healthcare situation in the past, an essential prerequisite for improving the quality of AKI management in the future. It needs to be mentioned that the study reports about healthcare variables in a certain region of Germany, and it is therefore not a representative for the country in general.

## 2. Methods

### 2.1. Patients

All patients treated at the Brandenburg Hospital between January and the end of December 2015 were screened. Data were extracted from the central database of the hospital. Before the study was started, the ethics committee of the university denied the necessity of any formal approvement of the project since the study was completely retrospective in nature. The term “nephrotoxic” was used for the following substances: nonsteroidal anti-inflammatory drugs (NSAID), aminoglycosides, vancomycin, and amphotericin B. Cardiovascular disease was defined as either drug-requiring arterial hypertension or as established coronary artery disease or chronic heart failure. Metabolic disease was diagnosed if subjects suffered from one or more of the following conditions: diabetes mellitus, obesity, and hyperuricemia. Lung disease was defined as either chronic obstructive lung disease or asthma or others (e.g., interstitial lung disease).

### 2.2. AKI Diagnosis

The diagnosis of AKI was made according to the 2012 published KDIGO clinical practice guideline for acute kidney injury [9]: an increase of the serum creatinine of at least 0.3 mg/dL (28.5 micromole/L) within 48 hours or (2) a 1.5-fold increase or more within 7 days.

A reliable documentation of urine output was only available for subjects treated at the ICU, which represented the minority. We therefore decided to exclude the parameter urine production as AKI definition criteria. In general, patients with preexisting chronic kidney disease (CKD) stages 3–5ND were also included, and subjects in stage CKD 5D were excluded from the study.

### 2.3. AKI Severity

AKI severity was classified according to the AKIN criteria [10].

### 2.4. Renal Replacement Therapy (RRT)

Renal replacement therapy was initiated if decided so by local specialists of internal medicine or anesthesiology. It was either performed in the hospital as continuous venovenous hemodialysis/hemodiafiltration using citrate as a regional anticoagulant or in other hospitals. If so, data on the procedures used were not available.

### 2.5. Renal Recovery

Renal recovery was defined by a decrease of the serum creatinine to the range before AKI became apparent. The initial range was defined as initial eGFR ± 10 ml/min.

### 2.6. Statistics

Prior to any analyzes, all groups were checked for normality using the Kolmogorov–Smirnov test. Two groups were compared with Student's *t*-test if normality was fulfilled or compared with the Mann–Whitney test if the results were not distributed normally. Three or more groups were compared with ANOVA. Results are either given as percentages or as mean ± SEM. Statistical significance was postulated if the *p* value was below 0.05.

## 3. Results

### 3.1. Patients' Characteristics

Between January and December 2015, 5,300 patients underwent treatment at the Brandenburg Hospital of the Brandenburg Medical School. The baseline characteristics of patients finally included in the study (patients that were diagnosed with AKI) are summarized in Table 1.

### 3.2. AKI Incidence and Etiology

**Figure 1:**
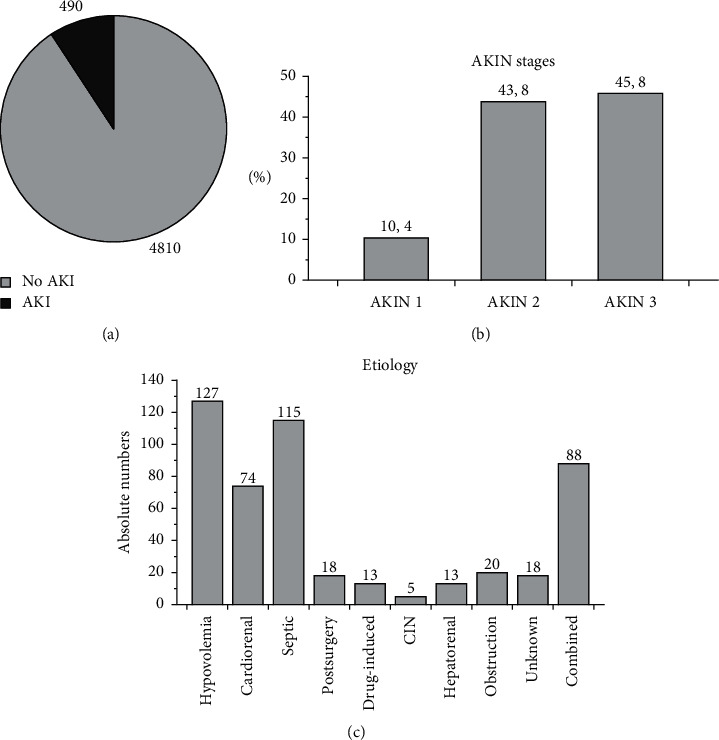
AKI incidence and etiology. (a) Incidence in all in-hospital subjects treated during the observation period. A total number of 5,300 patients were treated at the hospital between January and December 2015, 490 or 10.1% acquired acute kidney injury. (b) AKI severity according to the AKIN criteria. (c) Respective etiology.

According to the KDIGO criteria for AKI diagnosis, 490 subjects or 10.1% were diagnosed with acute kidney injury. Respective etiologies were hypovolemia (*n* = 127; 25.8%), cardiorenal syndrome (*n* = 74; 15%), sepsis (*n* = 115; 23.5%), postsurgery AKI (*n* = 18; 3.6%), drug-induced AKI (*n* = 13; 2.7%), contrast-induced nephropathy (*n* = 5; 1%), hepatorenal syndrome (*n* = 13; 2.7%), urinary tract obstruction (*n* = 20; 4%), combined (*n* = 88; 18%), and unknown (*n* = 18; 3.7%). According to the AKIN criteria (2), 50 (10.4%) developed stage 1, 210 (43.8%) presented stage 2, and 220 (45.8%) presented stage 3. The AKIN stages were not available in 2 subjects (Figure 1). 61% of all patients diagnosed with AKI had preexisting CKD of various etiology. The following parameters/characteristics were comparably distributed between AKIN stages 1 and 3: gender, age, duration of stay at the hospital (DOS), AKI onset (outpatient versus in-hospital), initial CRP, preexisting arterial hypertension, coronary artery disease, chronic heart failure, diabetes mellitus, obesity, and neoplasia. Other variables were not distributed homogenously between the AKIN stages: dialysis due to AKI, survival, renal recovery, initial serum creatinine in micromole/L, vasopressor therapy, and invasive ventilation. Detailed numbers and *p* values are given in Table 2.

### 3.3. Duration of In-Hospital Treatment

The mean duration of in-hospital treatment of all AKI subjects was 14.2 ± 1.8 days. If related to the AKIN stages, the respective durations were 13.5 ± 1.3 days (stage 1), 15.3 ± 1.2 days (stage 2), and 19.2 ± 3.9 days (stage 3). The periods did not differ in a significant manner (*p*=0.51).

### 3.4. Mortality

**Figure 2:**
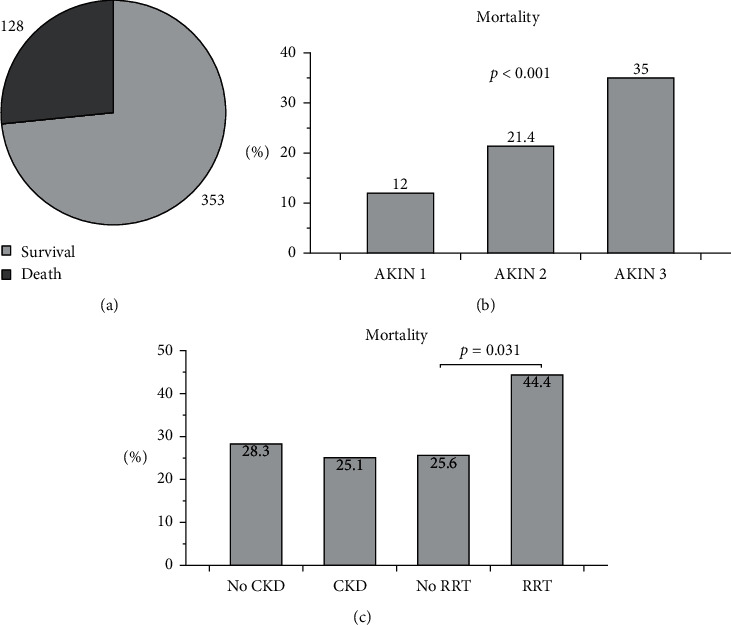
Mortality in all subjects and in different cohorts. (a) The total in-hospital mortality was 128 out of 481 patients (26%). (b) Mortality rates in relation to the AKIN stages, the risk of death continuously increased from stage 1 to 3. (c) Mortalities did not differ between subjects with and those without preexisting CKD. However, individuals undergoing RRT were at significantly higher risk to die than those without dialysis.

Data on mortality were missing in 10 subjects. The total in-hospital mortality of all AKI subjects was 26%. If related to the AKIN stages, the mortality rates were AKIN 1, 12%; AKIN 2, 21.4%; and AKIN 3, 35% (*p* < 0.001). If related to gender, 28.5% of all men and 24.3% of all women died during the stay at the hospital (*p*=0.29). The mortality rate of patients with acute deteriorated CKD was 25.1% in comparison to 28.3% in AKI subjects without preexisting chronic kidney dysfunction (*p*=0.44). Out of all subjects that received RRT in the Brandenburg Hospital, 44.4% died before discharge as opposed to 25.6% who did not receive dialyzes (*p*=0.031) (Figure 2). The following parameters/characteristics were comparably distributed between surviving and nonsurviving patients: gender, age, preexisting CKD, initial serum creatinine, initial CRP, preexisting arterial hypertension, coronary artery disease, chronic heart failure, diabetes mellitus, obesity, and neoplasia. Some other variables significantly differed between the two groups (always survivors vs. nonsurvivors): duration of stay at the hospital, AKI onset, dialysis due to AKI, vasopressor therapy, and invasive ventilation. Detailed information about numbers and *p* values is given in Table 3.

### 3.5. Renal Replacement Therapy

As pointed out, RRT-related data were only available in a limited fashion. Particularly, information about the procedures used for RRT in subjects transferred to other hospitals was unavailable. RRT-related information was available from 476 individuals (97%). Twenty-seven subjects received dialysis (5.6%) with 19 patients (3.9%) treated at the Brandenburg Hospital, and 8 patients (1.6%) were treated at other hospitals.

### 3.6. Renal Recovery

**Figure 3:**
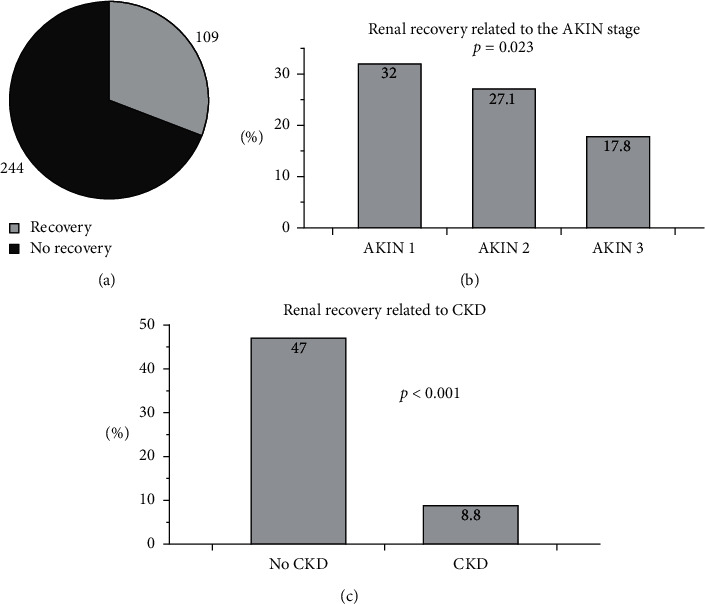
Renal recovery in all subjects and in different cohorts. (a) 109 out of 353 surviving patients (31%) recovered completely while 69% did not recover. (b), (c) The rates of recovery in relation to the AKIN stages (b) and to CKD (present/not present, c).

Analysis of renal recovery was exclusively performed in surviving subjects. Out of 481 patients, 353 individuals survived. Thirty-one percent (31%, *n* = 109) of surviving subjects showed (complete) renal recovery at the time of demission. Data on renal recovery were missing in 9 patients. Subjects with preexisting CKD recovered in 8.8%, and patients without preexisting chronic kidney disease recovered in 47% (*p* < 0.001). If related to the AKIN stages, recovery was observed in stage 1 (32%), stage 2 (27.1%), and stage 3 (17.8%) (*p*=0.023) (Figure 3). The following parameters/characteristics were comparably distributed between subjects with and those without recovery (always recovery vs. no recovery): gender, AKI onset, initial CRP, vasopressor therapy, invasive ventilation, obesity, and neoplasia. The distribution of several other parameters significantly differed between the two groups: age, duration of stay at the hospital, preexisting CKD, dialysis due to AKI, initial serum creatinine, preexisting arterial hypertension, coronary artery disease, chronic heart failure, and diabetes mellitus (numbers and *p* values, Table 3).

## 4. Discussion

At first, it needs to be mentioned that the findings reported in the study are not generalizable for the whole country. We intended to characterize healthcare-associated variables at one out of two university hospitals belonging to the first medical school of the federal state of Brandenburg. We evaluated the quality of renal care at a single university medical center prior to the implementation of a nephrology section.

Acute kidney injury remains a fundamental problem in hospitals worldwide, and early diagnosis and management are difficult, particularly since no therapeutic measures are available that allowed to accelerate kidney recovery or repair once acute damage has been evolved.

Several studies provided data about AKI incidences at the ICU. However, less information is available about the total (community-based) or even the overall in-hospital incidence of the syndrome. In 2006, Liangos and colleagues reported about the ARF (acute renal failure) incidence of subjects included in the NHDS database [11]. The latter contains discharge records of in-hospital patients from participating hospitals (∼1% of all discharges, US nationwide). ARF was defined by an increase of serum creatinine between 0.5 and 1.5 mg/dl. According to these criteria, the ARF incidence was reported 1.9%. Hsu and colleagues [12] published a large retrospective analysis, which was based on data provided by the so-called “Kaiser Permanente of Northern California,” a huge healthcare deliverer. The authors considered a period from January 1996 to December 2003, and more than 3.7 million subjects were included with a total person-year number of more than 15 million. AKI (herein: acute renal failure) incidences increased throughout the whole period. In the first two years, it was reported with 322.7 per 100,000 person-years. 6 years later, it had been increased to 522.4 per 100,000 person-years. If compared to newer reports, the incidences were quite low (0.32/0.52%). It needs to be emphasized that Hsu and colleagues did not employ the 2012 published KDIGO criteria for AKI diagnosis since their analysis was published in 2007. The threshold used for defining “acute renal failure” (AKI) was much higher than recommended in KDIGO. The introduction of these 2012 published criteria [9] changed the situation substantially. Zeng and colleagues [13] employed a threshold of 0.3 mg/dL over 48 hours and reported that de novo AKI had been evolved in 5,848 of 31,970 (18.3%) hospitalizations between January and December 2010. Our study identified an overall AKI incidence of 10.1%. In the study by Zeng et al. [13], prehospitalization outpatient serum creatinine was considered as well (if available), an approach which we were unable to implement. However, this might possibly explain the discrepant findings. The data by Zeng highlighted the fact that AKI occurs in more hospitalized individuals than reported in older studies by Liangos et al. [11, 12]. The reasons for such dynamics were discussed by Kohli as follows [14]: aging of the population in general, a higher degree of cumulative morbidity, more sensitive criteria for AKI diagnosis, the expansion of modifiable risk factors, and the implementation of electronic alert systems.

The overall AKI-associated mortality in our study was 26%. Thus, we were in line with data reported by Susantitaphong et al. in 2013 [15], who reported an average mortality rate of 23.9% in adults. In surviving patients, we identified longer in-hospital treatment periods, a higher rate of outpatient-acquired AKI, less frequently performed dialysis, and a less frequent use/need of vasopressors/for invasive ventilation. Surprisingly, several conditions such as arterial hypertension, coronary artery disease, chronic heart failure, diabetes mellitus, or obesity were diagnosed significantly more frequent in nonsurvivors. Khadzhynov and colleagues [8] in contrast reported the following parameters as mortality risk factors in AKI: diabetes mellitus, heart failure, neoplasia, higher age, and male gender. The mentioned study identified a mortality of 24.8% exclusively in AKIN stage 3 subjects, in which the mortality at our hospital reached 35%. We did not separately analyze outcome variables of AKI patients treated at the local intensive care unit (ICU). Under ICU conditions, mortality rates of 50% or even higher have been reported [4, 16].

Only 5.6% of the subjects included in our analysis received renal replacement therapy. If so, continuous treatment using citrate as anticoagulant was the preferred procedure (∼70%). Several studies provide information about RRT frequency in AKI. Overall incidences of ∼10% have been reported [1, 12, 17]. Thus, the RRT incidence in our hospital was lower. Since neither a dialysis unit nor a nephrologist was available during the screening period, and the necessity to perform dialysis may have not been realized in time or early enough. It was impossible to analyze whether subjects were transferred to another hospital for other reasons than to receive RRT. Therefore, some individuals that were dialyzed later during the course of the disease may have been missed.

Complete renal recovery was observed in 109 (31%) of all surviving AKI subjects. Pannu and colleagues [18] performed a population-based cohort study with more than 190,000 included subjects. Patients were recruited from a provincial claims registry (Alberta, Canada); the observational period was November 2002–December 2007. A total number of 7,014 subjects were diagnosed with AKI, and 62.7% survived until day 90. At day 150 after AKI diagnosis, 2,247 individuals showed renal recovery (∼51%). Recovery was defined as a serum creatinine value of within 25% of the baseline value and independence from renal replacement therapy [18]. We employed stricter criteria for defining renal recovery (initial eGFR ± 10 ml/min). This may partly explain the lower percentage of patients in our study.

In summary, our analyzes showed a comparably low overall AKI incidence. On the other hand, RRT was performed in less individuals, and renal recovery was observed less frequent than referenced by others.

### 4.1. Limitations

The first limitation of the study is the lack of prehospitalization creatinine values. It was almost impossible to decide whether AKI was already evolving at the time when patients entered the hospital or whether subjects with increased serum creatinine suffered from CKD instead. The same applies for CKD epidemiology. We stated that ∼60% of the subjects suffered from preexisting CKD. However, some of these individuals may have suffered from community-acquired AKI rather than from CKD.

Another weakness is the lack of follow-up data from the subjects.

It also needs to be mentioned that the general awareness for the necessity to perform RRT was most likely quite low in the past. We suspect that dialysis was performed exclusively in those subjects who fulfilled unavoidable criteria such as refractory hyperkalemia or hypervolemia.

Finally, the retrospective nature of the investigation is critical, since some individuals with preexisting kidney diseases may have been admitted to other hospitals initially. These and other bias are immanent to every retrospective investigation. Therefore, further information must and will be gathered in future prospective trials.

## 5. Key Messages

1. The Brandenburg Medical School was founded in 2014, and a nephrology section was opened in summer 2017. The aim of the study was to analyze AKI epidemiology and outcomes in one of two university hospitals belonging to the medical school in a period before summer 2017.
2. A total number of 5,300 patients were included in the analysis, and AKI was diagnosed in 490 subjects (10.1%). The in-hospital mortality was 26%. 5.6% received dialysis therapy, and renal recovery occurred in 31% of all surviving AKI subjects.
3. Both the AKI incidence and the frequency of dialysis were lower than that reported in the literature. However, fewer subjects recovered from AKI.
4. The discrepant findings possibly result from the lack of prehospitalization creatinine values, the lack of follow-up data, and a generally lower awareness for the need to perform renal replacement therapy in AKI.
5. The kidney-centered care of patients in Brandenburg requires improvement, particularly regarding the management of established AKI.

## Figures and Tables

**Table 1:** Patients' baseline characteristics.

Analyte	Result
Gender	Male 271; female 220
Age (years ± SEM)	73.4 ± 0.57
Duration of stay at the hospital (days)	15.02 ± 0.71
In-hospital survival (%)	74
Cardiovascular disease (%)	91.8
Metabolic disease (%)	50.6
Lung disease (%)	12.5
Preexisting chronic kidney disease (%)	61
Nephrotoxic medication (%)	2.7
Mechanical ventilation (%)	27.3
Vasopressor therapy (%)	31

**Table 2:** Distribution of epidemiological and morbidity-associated characteristics in AKIN stages I–III.

Risk factor	AKIN I (*n* = 50)	AKIN II (*n* = 210)	AKIN III (*n* = 220)	*p* value
Gender (%)	f: 58; m: 42	f: 46.2; m: 53.8	f: 41.8; m: 58.2	0.11
Age (mean years ± SEM)	71.5 ± 2.1	74.5 ± 0.8	72.7 ± 0.9	0.18
DOS (mean days ± SEM)	13.7 ± 1.4	15.3 ± 1.2	14.2 ± 0.8	0.69
Preexisting CKD (%)	46	68,3	58,6	**0.007**
AKI onset (outpatient vs. in-hospital, %)	o: 54.2; i: 45.8	o: 64.7; i: 35.3	o: 63.8; i: 36.2	0.38
Dialysis due to AKI (%)	0	0,5	12	**<0.001**
Survival (%)	88	78,6	65	**<0.001**
Renal recovery (%)	32	27,1	17,8	**0.023**
Creatinine initially (micromole/L ± SEM)	124 ± 6.4	169 ± 4.6	329 ± 17.4	**<0.001**
CRP initially (mg/L ± SEM)	61.2 ± 11.3	91.5 ± 7.7	100.9 ± 7.6	0.07
Vasopressor (%)	16	25,2	39,3	**<0.001**
Invasive ventilation (%)	16	24,3	32,9	**0,02**
Arterial hypertension (%)	84	90	90	0,42
Coronary artery disease (%)	34	36,7	30,5	0,39
Chronic heart failure (%)	38	45,2	38,2	0,29
Diabetes mellitus (%)	36	36,2	38,6	0,85
Obesity (%)	18	17,1	12,7	0,37
Neoplasia (%)	18	31,6	30,6	0,15

The values in bold represent statistically significant results.

**Table 3:** Distribution of epidemiological and morbidity-associated characteristics in surviving versus nonsurviving subjects and in those with versus without recovery of kidney function (renal recovery).

Risk factor	Survival (yes or yes vs. no)	*p* value	Renal recovery (yes or yes vs. no)	*p* value
Gender (%)	f: 46.7; m: 53.3	0.29	f: 47.8; m: 5.2	0.54
Age (mean years ± SEM)	72.8 ± 0.6 vs. 74.9 ± 1.1	0.12	69.3 ± 1.2 vs. 74.6 ± 0.6	**<0.001**
DOS (mean days ± SEM)	16 ± 0.8 vs. 10.6 ± 0.9	**<0.001**	20.6 ± 2.2 vs. 12.8 ± 0.5	**<0.001**
Preexisting CKD (%)	62.6 vs. 58.7	0.44	23.2 vs. 73.5	**<0.001**
AKI onset (outpatient vs. in-hospital, %)	o: 66.7 vs. i: 53.7	**0.01**	o: 69.4 vs. i: 61.4	0.13
Dialysis due to AKI (%)	4.3 vs. 9.4	**0.03**	0.9 vs. 6.9	**0.01**
Creatinine initially (micromole/L ± SEM)	239 ± 11.2 vs. 236 ± 14.9	0.88	180 ± 12.7 vs. 256 ± 11.1	**<0.001**
CRP initially (mg/L ± SEM)	86.7 ± 5.6 vs. 108 ± 10.5	0.5	100 ± 10 vs. 89 ± 5.7	0.35
Vasopressor (%)	23 vs. 52.3	**<0.001**	30.4 vs. 31.1	0.88
Invasive ventilation (%)	20.5 vs. 46.1	**<0.001**	30.4 vs. 26.4	0.41
Arterial hypertension (%)	89.5 vs. 89.1	0.88	84.1 vs. 91	**0.03**
Coronary artery disease (%)	31.4 vs. 39.8	0.08	23 vs. 37	**0.006**
Chronic heart failure (%)	40.8 vs. 43	0.66	27.4 vs. 45.7	**<0.001**
Diabetes mellitus (%)	34.8 vs. 43.8	0.07	28.6 vs. 39.7	**0.03**
Obesity (%)	16 vs. 15.6	0.86	15.9 vs. 14.9	0.8
Neoplasia (%)	29 vs. 31.2	0.64	30 vs. 29.6	0.92

The values in bold represent statistically significant results.

## Data Availability

The data used to support the findings of this study are available from the corresponding author upon request.
